# Regeneration of segmental defects in metatarsus of sheep with vascularized and customized 3D-printed calcium phosphate scaffolds

**DOI:** 10.1038/s41598-020-63742-w

**Published:** 2020-04-27

**Authors:** Luciano Vidal, Carina Kampleitner, Stéphanie Krissian, Meadhbh Á Brennan, Oskar Hoffmann, Yago Raymond, Yassine Maazouz, Maria-Pau Ginebra, Philippe Rosset, Pierre Layrolle

**Affiliations:** 1 Inserm, UMR 1238, PHY-OS, Bone sarcomas and remodelling of calcified tissues, Faculty of Medicine, University of Nantes, Nantes, 44035 France; 2 University of Vienna, Department of Pharmacology and Toxicology, Vienna, 1090 Austria; 3 Department of Trauma and Orthopaedic Surgery, University Hospital of Tours, University of Tours, Tours, 37000 France; 4 John A. Paulson School of Engineering and Applied Sciences, Harvard University, Cambridge, MA 02138 USA; 5 Universitat Politècnica de Catalunya, Department of Materials Science and Metallurgical Engineering, Group of Biomaterials, Biomechanics and Tissue Engineering, Barcelona, 08019 Spain; 6 Universitat Politècnica de Catalunya, Barcelona Research Centre for Multiscale Science and Engineering, Barcelona, 08019 Spain; 7 Mimetis Biomaterials, Cerdanyola del Vallès, Barcelona, 08290 Spain; 8 Institute for Bioengineering of Catalonia, Barcelona Institute of Science and Technology, Barcelona, 08036 Spain; 9 Platform CIRE, Surgery and Imaging for Research and Education, INRA, Nouzilly, 37380 France

**Keywords:** Translational research, Biomedical materials

## Abstract

Although autografts are considered to be the gold standard treatment for reconstruction of large bone defects resulting from trauma or diseases, donor site morbidity and limited availability restrict their use. Successful bone repair also depends on sufficient vascularization and to address this challenge, novel strategies focus on the development of vascularized biomaterial scaffolds. This pilot study aimed to investigate the feasibility of regenerating large bone defects in sheep using 3D-printed customized calcium phosphate scaffolds with or without surgical vascularization. Pre-operative computed tomography scans were performed to visualize the metatarsus and vasculature and to fabricate customized scaffolds and surgical guides by 3D printing. Critical-sized segmental defects created in the mid-diaphyseal region of the metatarsus were either left empty or treated with the 3D scaffold alone or in combination with an axial vascular pedicle. Bone regeneration was evaluated 1, 2 and 3 months post-implantation. After 3 months, the untreated defect remained non-bridged while the 3D scaffold guided bone regeneration. The presence of the vascular pedicle further enhanced bone formation. Histology confirmed bone growth inside the porous 3D scaffolds with or without vascular pedicle inclusion. Taken together, this pilot study demonstrated the feasibility of precised pre-surgical planning and reconstruction of large bone defects with 3D-printed personalized scaffolds.

## Introduction

Bone is a dynamic tissue that possesses the intrinsic capacity to heal within 6–8 weeks after immobilization of a fracture. However, there are some conditions in which bone regeneration is delayed, compromised or beyond the physiological healing potential^1,2^. Notably, the successful repair of large bone defects caused by trauma, tumor resection or disease remains a clinical challenge for orthopedic and plastic surgeons and often requires additional treatments. Autologous bone grafting is still considered the gold standard treatment due to its osteoconductive, osteoinductive and osteogenic properties. This procedure necessitates harvesting the patient’s own bone and subsequently transplanting it to the defect site. Bone can be taken from several areas e.g., iliac crest or fibula, depending on the severity and amount needed for reconstruction of the defect. Nevertheless, the amount of bone is limited and the procedure adds morbidity at the harvesting site. Therefore, this surgical procedure is often associated with complications such as infection, hematoma, postoperative pain, and muscular and neural damage. Transplantation of vascularized bone also requires complex microsurgery to adapt to both the local vasculature and skeleton^3,4^. Other procedures for large bone regeneration are the Masquelet’s induced membrane, the Illizarov’s distraction or advanced therapies with bone morphogenetic proteins or culture expanded bone marrow mesenchymal stem cells. However, these alternatives have inherent disadvantages including morbidity, surgical procedures, costs and safety concerns^5–10^.

For several decades, researchers and clinicians have attempted to develop a safe and effective alternative to autologous bone grafting for the regeneration of large bone defects. Among them, synthetic calcium phosphate biomaterials that resemble the inorganic phase of bone have proven to be biocompatible and osteoconductive. Most commercially available calcium phosphate-based bone substitutes are composed of either hydroxyapatite (HA), β-tricalcium phosphate (β-TCP), or a mixture of both, termed biphasic calcium phosphate (BCP). They are made at high sintering temperatures and are generally used as granules or porous blocks. Although these bioceramics share some compositional similarities with bone minerals, the conventional processes employed to manufacture them limit the possibilities to tune their pore architecture, often lacking interconnections and hindering their osteogenic potential to support healing of large bone defects^11–14^. Recently, biomimetic calcium phosphates consisting of calcium-deficient hydroxyapatite (CDHA) that can be manufactured at ambient temperature have been developed and have shown to have enhanced surface area and promote osteogenic differentiation and bone healing compared to their sintered counterparts^15,16^.

Three-dimensional (3D) printing permits the production of customized scaffolds and surgical guides based on the patient’s anatomy from computed tomography (CT) scans that are essential for surgical planning, precise resection and accurate bone reconstruction. CDHA inks have been developed to manufacture 3D scaffolds using a layer-by-layer deposition, also known as 3D-microextrusion. The advantages of this 3D-printed biomaterial are the structural and compositional features that closely resemble the mineral phase of bone compared with conventional bioceramics, and the ambient temperature manufacturing process that offers the possibility of reinforcing the structure with polymers or incorporate drugs. Furthermore, 3D printing allows accurate control of construct shape and their interconnected porosity favoring body fluids permeability, cell invasion, vascularization and bone ingrowth, making these 3D scaffolds promising therapeutic alternatives to treat large bone defects^15,17–19^.

Another essential aspect of bone transplantation is vascularization. The development of a vascular network is crucial for the exchange of nutrients, minerals and soluble molecules which are important during the repair process and ensure the viability of the bone graft. Lack of vascularization causes inner tissue necrosis and results in graft failure, particularly in large bone defects. In this respect, the interconnected porosity of the 3D scaffold facilitates the formation of new blood vessels throughout the entire material. Recent studies have attempted to enhance vascularization by either pre-vascularizing the construct before implantation or by adding angiogenic growth factors such as vascular endothelial growth factor (VEGF) or platelet-derived growth factor (PDGF) during the grafting procedure. Although these approaches showed promising results, novel strategies are required to further enable vascularization, in particular for large bone defects^12,20–22^.

This work aims to investigate the feasibility of a one-step surgical skeletal reconstruction and regeneration of large bone defects in a relevant pre-clinical animal model. We hypothesize that the application of a 3D-printed CDHA scaffold with pre-defined shape and interconnected porosity combined with a local and axial vascular pedicle will improve bone regeneration.

## Results

### Pre-operative computed tomography (CT) for designing customized surgical guides and 3D scaffolds

**Figure 1:**
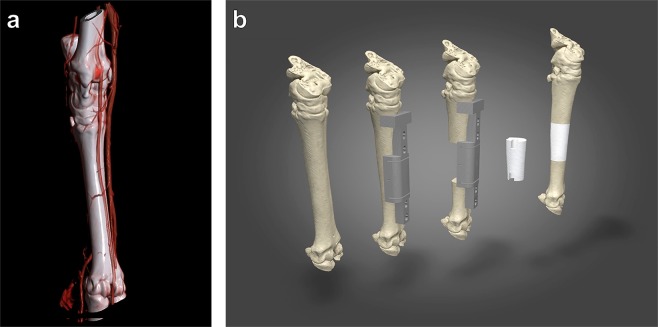
Design of the surgical cutting guide for the creation of a segmental mid-diaphyseal defect and the customized 3D scaffold. (**a**) Pre-operative CT scans were taken one month before surgery and 3D reconstructed. The image visualizes the metatarsal bone and the local vasculature. (**b**) Schematic diagram demonstrates the operating principle of the surgical guide and the filling of the bone defect (35 mm in length) with a customized 3D scaffold with a groove for axial vascularization.

One month before surgery, a CT angioscan of the metatarsus of sheep was performed to visualize the local vasculature and bone (Fig. 1a). This 3D reconstruction shows that three main blood vessels were present along the metatarsal bone, while the lateral plantar artery was selected for axial vasculature of the 3D scaffolds. As shown in Fig. 1b, a customized surgical guide was designed to assist for the creation of a critical-sized segmental diaphyseal defect (length: 35 mm) in the metatarsus of sheep, and for drilling the holes for the osteosynthesis screws to fix the plate that stabilizes the fracture. This pre-operative CT scan was also used to manufacture a customized 3D scaffold with interconnected porosity that fitted the defect and a groove to incorporate the axial vascular pedicle.

### Physicochemical characterization of the 3D-printed calcium phosphate scaffolds

**Figure 2:**
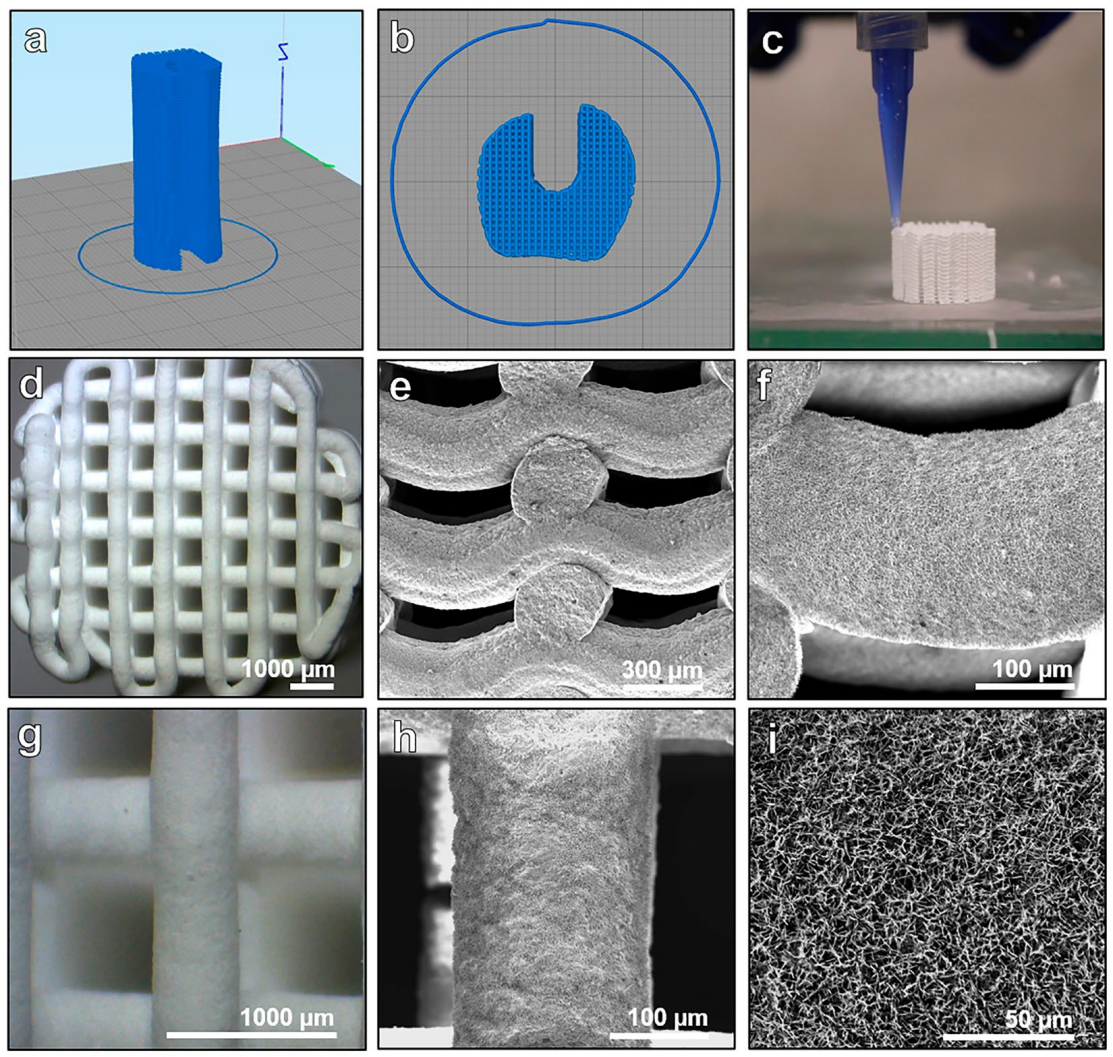
Fabrication and structure of a 3D-printed customized calcium phosphate scaffold. (**a–c**) Design and printing of a calcium phosphate scaffold by 3D-microextrusion. (**d–i**) Optical and scanning SEM images of the 3D-printed scaffolds. Images were taken at different levels of magnification to visualize the structure and surface of the material.

As shown in Fig. 2, the 3D scaffolds were designed with interconnected porosity and a groove to accommodate the axial vascular pedicle. The 3D scaffolds were then printed by using a robocasting device with a syringe containing the calcium phosphate paste coupled to a nozzle. A vascular plug with rounded edges was used for retaining the axial vascular pedicle inside the 3D scaffold (Supplementary Fig. 1). The porosity had an orthogonal rectilinear pattern with alternate crisscrossed layers rotated 90° relative to the previous layer creating an interconnected pore network mesh. The microstructure of the 3D scaffolds, as observed by scanning electron microscopy (SEM), consisted of an entangled network of nanosized needle-like hydroxyapatite crystals.

The physicochemical characteristics of the 3D scaffolds are reported in Table 1. After setting, the 3D scaffolds were composed of 80.3% of CDHA, 18.7% β-TCP and no presence of its alpha polymorph (α-TCP). The main body presented an inter-strand gap of 703.2 ± 23.9 µm and a filament width of 489.7 ± 9.7 µm in line with the 410 µm-nozzle used for 3D printing, whereas the scaffolds printed with the 311 µm-nozzle-setup presented an inter-filament separation of 529.4 ± 25.3 µm and a filament width of 364.2 ± 11.9 µm. According to the mercury intrusion porosimetry (MIP) analysis, the scaffold presented 81.03% of open porosity of which 59.52% correspond to pores with entrance sizes larger than 10 µm (macro-pores) and the remaining 21.52% corresponding to pores with entrance sizes smaller than 10 µm (micro/nano-pores). The specific surface area of the 3D scaffolds was 22.1 m^2^/g in agreement with their microstructure. The uniaxial compression testing assays resulted in similar values for both conditions: the scaffolds printed with the 410 µm-nozzle-setup and 311 µm-nozzle-setup had an ultimate compressive strength of 2.10 ± 0.30 and 2.45 ± 0.53 MPa respectively.

**Table 1:** Physicochemical properties of the 3D scaffolds.

3D Scaffold	Shape size (mm)	Crystal phase composition	Filament width (µm)	Pore size (µm)	Porosity %	Specific surface area (m^2^/g)	Ultimate compressive strength (MPa)
Main body printed with 410 µm-nozzle	Cylinder (H × Ø): 35 × 15 Groove (D × W): 7 × 5	80.3% CDHA 18.7% β-TCP 0% α-TCP	489.7 ± 9.7	703.2 ± 23.9	Total: 81.03 Macro (>10 µm): 59.52 Micro (<10 µm): 21.52	22.1	2.10 ± 0.30
Vascular plug printed with 311 µm-nozzle	Trapezoid (L × H): 30 × 8		364.2 ± 11.9	529.4 ± 25.3			2.45 ± 0.53

### Animal welfare and surgical procedure

**Figure 3:**
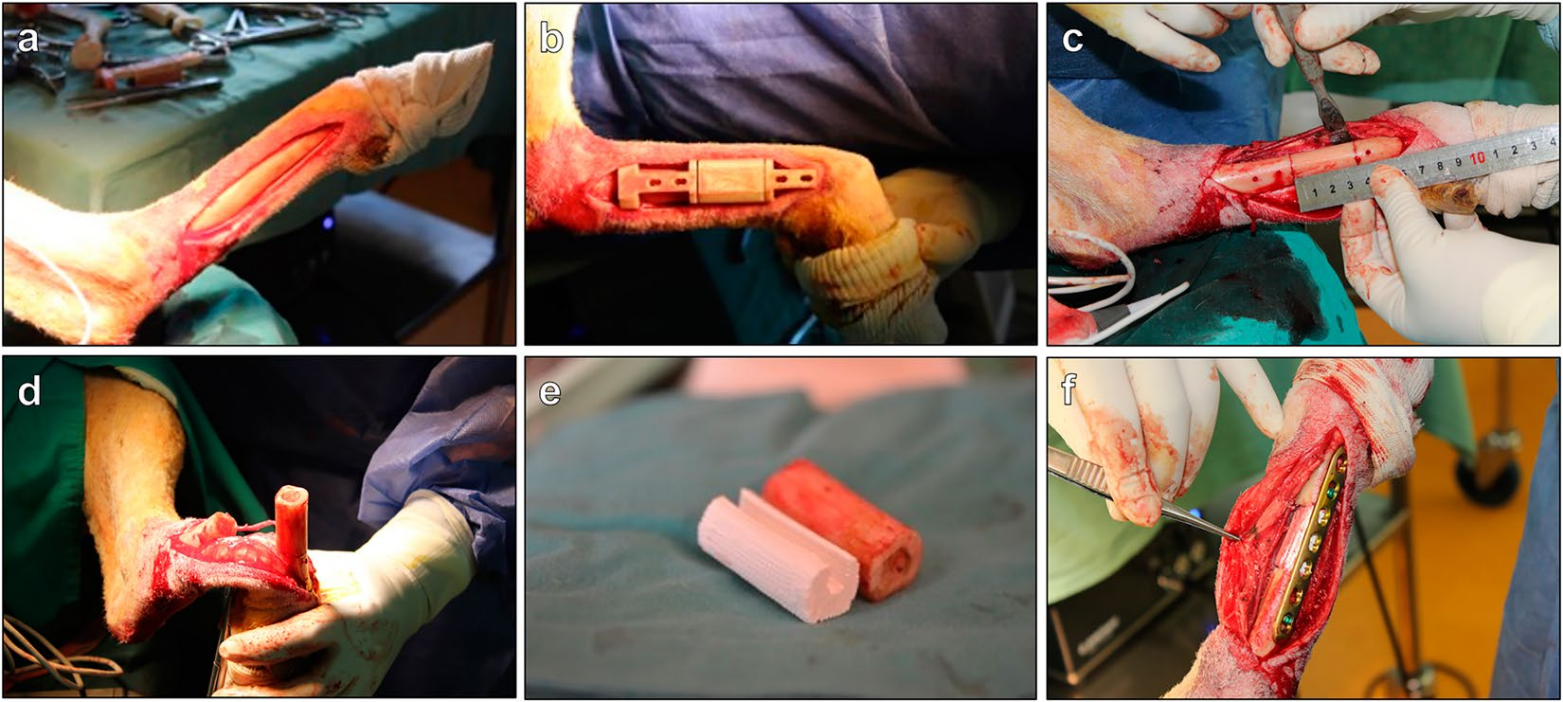
Creation of a critical sized segmental defect in sheep. Photographs of the surgical steps showing (**a**) the metatarsal bone, (**b**) the surgical guide used to create the bone defect, (**c**) the creation of a mid-diaphyseal segmental defect measuring 35 mm in the metatarsal bone, (**d**) the vascular pedicle used for the axial vascularization to the 3D-printed scaffold, (**e**) the customized shape of the 3D-printed scaffold and (**f**) the final result with the osteosynthesis plate fixation.

All sheep survived the operative intervention without any early or long-term post-operative complications. Figure 3 demonstrates the surgical procedure using the pre-designed customized surgical guide and the 3D-printed scaffold. The application of the surgical guides supported the creation of a segmental critical-sized defect of 35 mm at the mid-diaphyseal level of the metatarsal bone and directed the drilling for the osteosynthesis plate. This controlled drilling allowed an accurate positioning during the perforation and avoided damage to the surrounding soft tissues. In addition, they helped to maintain the reproducibility in all the procedures. The lateral plantar artery (Arteria plantaris lateralis) that was isolated and passed through the 3D-printed scaffold to improve vascularization (3D scaffold + pedicle group) is shown in Fig. 3d. Figure 3e indicates identical anatomical shapes of the customized 3D-printed calcium phosphate scaffolds made by reverse engineering from CT scans compared to the bone resected at the day of surgery. The final reconstruction with the axial vascular pedicle passing through the anatomical 3D scaffold held by the fixation plate and screws is illustrated in Fig. 3f.

### *In vivo* evaluation of 3D-printed calcium phosphate scaffolds

**Figure 4:**
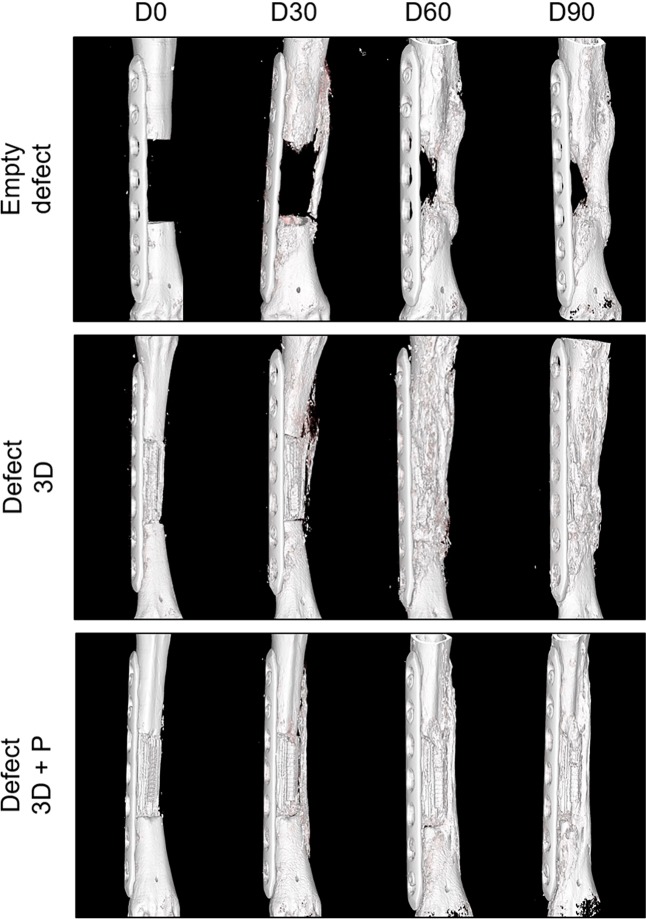
3D bone reconstructions of metatarsal defects in sheep. CT scans are shown post-surgery (D0) and after 30, 60 and 90 days (D30, D60, D90) of healing. Treatment groups included the empty defect (control), a defect filled with the 3D-printed scaffold (Defect 3D), and a defect treated with the 3D-printed scaffold and the axial vascular pedicle (Defect 3D + P) (n = 1).

To study the ability of the scaffold to regenerate a large bone defect, bone repair was examined by *in vivo* CT scans over a healing period of 3 months (day 0, 30, 60 and 90). As shown in Fig. 4, some bone regeneration occurred from the edges of the left empty defect on the side opposite to the osteosynthesis plate as early as D30. It indicated the endogenous healing potential of the mid-diaphyseal defect created in the metatarsus of sheep. In contrast, the defect filled with the 3D-printed scaffold and the 3D-printed scaffold combined with the vascular pedicle showed enhanced bone formation over the 3-month healing period. The scaffolds appeared well-integrated into the defect and surrounded by newly formed bone tissue. The origin of the bone regrowth was similar to that of the empty defect group and predominantly started from the external lateral side, opposite to the osteosynthesis plate. To monitor and control the durability of the vascular pedicle and vascular patency, angioscans were conducted by injecting an iodine contrast agent during the CT scan at day 30, 60 and 90. The vascular patency of the vascularized 3D-printed scaffold group was maintained for the 3 months of the study (Supplementary Fig. 2).

**Figure 5:**
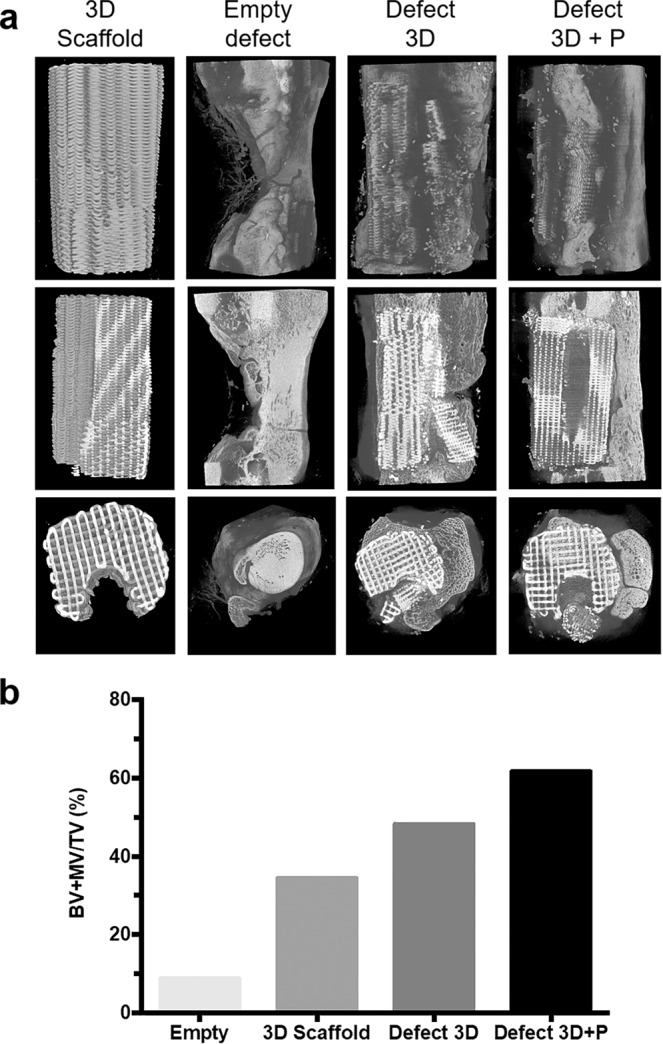
MicroCT images of treated and untreated segmental defects in sheep. (**a**) MicroCT 3D reconstructions of metatarsal defects in sheep 90 days post-surgery. Images are presented for the scaffold before implantation (3D Scaffold), empty defect, defect filled with the 3D-printed scaffold (Defect 3D) and the defect filled with the 3D-printed scaffold associated with a vascular pedicle (Defect 3D + P). (**b**) Percentage of bone volume + material volume/total volume (BV + MV/TV) for the different treatment groups. The percentage of BV + MV/TV was calculated in the area of interest and compared to the microCT of the 3D scaffold taken before implantation (n = 1).

Micro-computed CT (microCT) scans calculations confirmed that the 3D scaffold before implantation had a rectangular interconnected porosity with a material volume/total volume (MV/TV) of 34.4% (n = 1), giving a macroporosity of 65.6%, in good agreement with the macroporosity of 59.5% measured by MIP (Fig. 5 and Table 1). Additionally, *ex vivo* high-resolution microCT was performed at the endpoint of the study (day 90) (Fig. 5). The left empty metatarsal defect had limited bone regeneration with a BV/TV value of 8.7% (n = 1). In contrast, the metatarsal defects filled with the customized 3D scaffolds and *a fortiori* in combination with the vascular pedicle showed a higher bone content, with BV + MV/TV values of 48.2% (n = 1) and 61.7% (n = 1), respectively.

**Figure 6:**
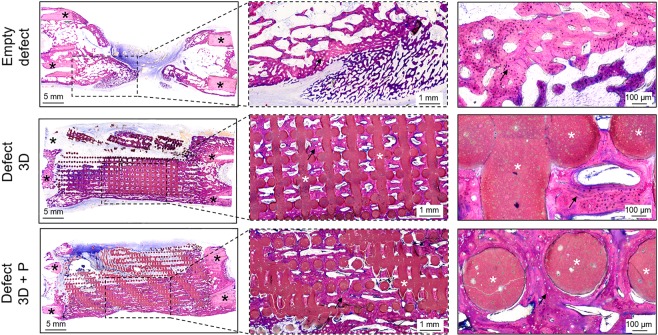
Histological evaluation of bone repair. Representative undecalcified histological thin ground sections were prepared in the longitudinal plane and stained with Levai-Laczko dye 90 days post-surgery (n = 1). Photomicrographs are presented at three different magnifications. The black asterisk denotes the host bone (old bone) stained in light purple, whereas the black arrow demonstrates newly formed bone tissue visible in dark purple. The white asterisk highlights the 3D scaffold (n = 1).

As illustrated in Fig. 6, the histological evaluation corroborated the CT and microCT findings. The empty defect was mainly filled with fibrous tissue and demonstrated limited bone tissue formation from the defect edges, whereas metatarsal bone defects filled with a customized 3D-printed scaffold or the 3D-printed scaffold combined with the axial vascular pedicle demonstrated bone ingrowth into the 3D structure of the biomaterial. At higher magnification, bone tissue containing osteocytes was present in contact with the 3D scaffold material. Osteocytes were more prevalent in the 3D-printed scaffold in combination with a vascular pedicle, indicating the formation of mature bone tissue in this latter group.

## Discussion

Bone is the second most transplanted tissue after blood transfusion. Vascularization of the transplanted bone graft is essential for favorable clinical outcomes. Indeed, bone cells are located near blood vessels to ensure access to oxygen and to receive the necessary nutrients to regenerate tissues. It has been shown that with a sufficient amount of vascularization these bone cells can differentiate into osteocytes and thus regenerate bone tissue^23–25^. However, the current transplantation of vascularized bone requires complex microsurgery to reconnect vasculature and to fit the anatomy of a defect, as well as adding morbidity at the harvesting site.

Tissue engineering is potentially a promising alternative to autologous vascularized bone grafting for the reconstruction of large skeletal defects^26–29^. Numerous pre-clinical studies have demonstrated that the combination of mesenchymal stem cells (MSCs) with BCP biomaterials can induce bone formation and facilitate the healing of critical size defects^30–33^. Some clinical trials have demonstrated the feasibility of amplifying autologous MSCs in culture from a bone marrow sample and associating them with BCP biomaterial to regenerate unconsolidated fractures or small defects^8,34^. However, these tissue engineering techniques do not yet allow the regeneration of large bone defects due to lack of adequate vascularization inhibiting cell survival and bone healing^35,36^. In this context, several teams have proposed the pre-vascularization of the bone filling material by implantation in an ectopic site before transplanting it into the defect to be reconstructed. Proof of concept has been demonstrated at the pre-clinical level by ectopic implantation in the ewe of a chamber containing the BCP biomaterial and an arteriovenous fistula (vascular loop). After 6 and 12 weeks of implantation, abundant microvasculature was observed in the chamber contents^37–42^. Large defects at the mandibular level have been successfully reconstructed in a few patients using this technique of transplanting a pre-vascularized biomaterial from an intramuscular site^43,44^. In these studies, the pre-vascularization of the biomaterial appears to dominate for cell survival and bone regeneration. Recently, *Charbonnier et al*. published a new arteriovenous bundle technique study enclosing a vascular pedicle, just the vein, passing centrally through a 3D-printed calcium phosphate scaffold^45^.

To our knowledge, our contribution is the first work in which, a pre-surgical CT angioscan allowed to create a truly personalized biomaterial scaffold that has the same anatomical shape as the defect. Moreover, with the pre-surgical CT angioscan, we were able to study the vasculature in the defect area to be reconstructed, allowing us to evaluate the possibility of isolating a pedicle composed of a peripheral artery and an adjacent vein. This pedicle was dissected from the surrounding tissues and passed through the implantable 3D-printed scaffold in order to provide vascularization without adding laborious microsurgery. This pilot study in sheep demonstrates the feasibility of fabricating customized 3D scaffolds and surgical guides based on CT scans that are essential for surgical planning, precise resection and accurate anatomical fitting of the 3D scaffold with anatomy. It is important to emphasize that in our work we performed a personalized large bone defect reconstruction with a 3D-printed scaffold associated with a local vascular pedicle in a one-step surgery. Furthermore, 3D printing did not only allow control over the shape of the 3D scaffolds in order to accurately fit the anatomy of defects, but also permitted the design and production of an interconnected porosity favorable for body fluid permeability, vasculature and bone ingrowth.

Some limitations of the current pilot study include firstly that some bone tissue regeneration was observed in the empty defect that has been previously described as a critical size bone defect^37^. We consider that this regenerated bone tissue was developed from the vascularized membrane that is constituted around the metatarsal bone and in the subcutaneous tissue which was not resected in this first study. To investigate this, we resected this membrane and our empty defect did not regenerate any bone tissue during the first 3 months (Supplementary Fig. 3). Secondly, the 3D scaffolds, even though they supported bone ingrowth, had insufficient mechanical properties in comparison with those of native bone tissue. It will be necessary to provide better mechanical reinforcement, through for example, the addition of polymers. Although feasibility is demonstrated in a clinically relevant large animal model, this is a pilot study with only one animal per group, limiting statistical comparison. A further study in a larger population of animals is needed to assess the efficacy of this innovative approach and to compare bone regeneration and vascularization with or without an axial vascular pedicle in the 3D scaffolds.

To conclude, this pilot study that used pre-operative medical imaging, 3D printing of customized surgical guides and 3D scaffolds with a local and axial vascularization, might constitute a new approach for bone regeneration with a synthetic bone graft substitute. This treatment strategy will potentially mimic the currently most employed autologous technique in the reconstruction of large bone defects, the fibula flap, but without the generation of a second surgical site and associated disadvantages.

## Methods

### Ethical approval

Ethical approval for animal experimentation was obtained from the local ethics committee (Comité d’ethique en experimentation animale Val de Loire, CEEA19, France, ref. number 9235) and the French Ministry of Superior Education, Research and Innovation on February 28, 2018, by following the European Guidelines for Animal Care Directive 2010/63/EU.

### Animals

The animal study was performed at the CIRE platform (agreement number: A371754; INRA, Nouzilly, France) that is fully equipped with an operating theatre, housing and medical imaging.

The 3-year old female Ile de France sheep (average weight of 65 kg) were supplied from the INRA breeder and were housed in boxes at the experimental facility. The animals were fed with straw and received water ad libitum. The animals were acclimated for a minimum of 2 weeks prior to the first intervention.

### Pre-operative computed tomography (CT)

One month before surgery, the sheep were placed under general anesthesia by intravenous injection of ketamine (20 mg/kg, Kétamidor^®^, Axience, France) and xylazine (0.05 mg/kg, Rompun^®^ 2%, Bayer, France). Animals were intubated and put under gaseous relay with 3% isoflurane (Isoflurin^®^, Axience, France) carried by 100% oxygen. The sheep was then placed in the right lateral decubitus position on the CT scanner table (Dual-source 64-slice spiral Somatom^®^, Siemens, France) with the left limb immobilized. Acquisition of the bone metatarsus on the left leg was performed at 300 mAs and 140 kV with a section of 0.6 mm generating 748 DICOM images in approximately 15 s. To visualize the vasculature, 80 ml of iodine contrast agent (350 mg I/ml; Omnipaque™, GE Healthcare, France) was injected in the jugular vein via a catheter (5 ml/s). Acquisitions were performed at 80, 120, 140 and 160 s post-injection of the contrast agent. CT scans were reconstructed and analyzed by using the imaging software syngo.via, (Siemens, France).

### Design and fabrication of customized surgical guides using 3D printing

The customized surgical guide was designed to assist for the creation of a critical-sized segmental diaphyseal defect (length: 35 mm) in the metatarsus of sheep, and for drilling the holes for the osteosynthesis screws to fix the plate that stabilizes the fracture. Its main objective was to hold precisely the surgical oscillating blade and the drilling bur. To fabricate the surgical guide, DICOM images from the pre-operative CT scans of the metatarsi and the titanium osteosynthesis plates (LCP 3.5, 7 holes, L98 mm, Johnson & Johnson Medical) were converted to 3D stereolithography (STL) files using an open-source software (3D Slicer). Thereafter, the surgical guide was planned in the CAD program (Cinema 4D, Maxon, Germany). A phantom of the 3D scaffold and the metatarsus were produced in the same manner to examine the anatomical shape and size of the design (Fig. 1). The STL files of the different components (customized surgical guide, 3D scaffold and metatarsus) were translated into a printable g-code (3D printing software, Ultimaker Cura 3.0, Ultimaker, The Netherlands). The customized surgical guide was printed with nylon filament (2.85 ± 0.05 mm, Ultimaker) that sustained autoclave sterilization using a commercial 3D printer (Ultimaker 3 extended, Ultimaker). The metatarsus and 3D scaffold were printed with thermoplastic poly-lactic acid (PLA, 2.85 ± 0.10 mm, Ultimaker) in order to verify anatomical precision fitting to anatomy prior to surgery.

### 3D printing of the customized calcium phosphate scaffolds

The customized 3D calcium phosphate scaffolds (MimetikOss 3D, Mimetis Biomaterials S.L., Spain) were designed by reverse engineering from CT scans of the metatarsus of sheep and fabricated by robocasting, also known as direct ink writing, as previously published^15,17^. Briefly, a CDHA self-setting ink was prepared by mixing 35 wt% aqueous solution of poloxamer 407 (P2443 – Pluronic, F-127, Sigma-Aldrich, Missouri, USA) and α-TCP (Innotere GmbH, Radebeul, Germany) powder at a ratio of 0.5 g/g. 3D scaffolds were printed by using a robocasting device (Heavy Duty Paste Extruder, BCN3D Technologies, Barcelona, Spain) with a syringe (Optimum^®^ Syringe, Nordson EFD, U.S.A) containing the paste coupled to a tapered dispensing tip (SmoothFlow Tapered Tips, Nordson EFD) mounted on the machine. The 3D scaffold consisted of a two-part assembly as displayed in Supplementary Figure 1. The main body was a cylinder (length: 35 mm, diameter: 15–17 mm) with a central groove of 5 mm in width and 7 mm in depth for passing through the axial vascular pedicle. A vascular plug (length: 30 mm, height: 8 mm) with rounded edges was used for retaining the vascular pedicle inside the 3D scaffold. The geometry of the pores had an orthogonal rectilinear pattern with alternate crisscrossed layers rotated 90° relative to the previous layer creating an interconnected pore network mesh. Two different printing setups were defined for the two parts of the assembly. For the main body, we used a nozzle diameter of 410 µm (410 µm-nozzle-setup, 22 gauge) and for the vascular plug, a nozzle diameter of 311 µm (311 µm-nozzle-setup, 24 gauge). The filament width was set to 370 µm and 490 µm respectively. The printing process was followed by a hydrothermal treatment for hardening as described elsewhere^46^. Subsequently, the samples were packaged in a double sterilization pouch and sterilized by autoclaving.

The dimensions of the customized 3D scaffolds, strand width and height and pore size in the X/Y plane and in the Z direction were measured with the combination of an optical microscopy (Luxeo 4D Digital Stereozoom Microscope, Labomed Europe, Capelle aan den Ijssel, The Netherlands) and an image analysis software (Fiji, ImageJ) as previously described^46^. A total of 20 measurements on 3 different samples of each printing setup were carried out. The microstructure of the scaffold was observed by SEM (Phenom XL, Phenom-World B.V., ThermoFisher Scientific, Eindhoven, The Netherlands). The phase composition of the scaffolds was assessed by X-ray diffraction (D8 Advance, Bruker, MA, USA) The inter-strand porosity distribution (range from 0.006 to 100 µm) was measured by mercury intrusion porosimetry (MIP) (AutoPore IV Micromeritics, GA, USA). This technique was combined with helium psychometry (AccuPyc 1330, Micromeritics, GA, USA) and apparent density measurements, as described elsewhere^47^, in order to obtain an analysis of the porosity percentages in the scaffolds. The mechanical properties (ultimate compressive strength) were investigated using a universal testing machine (Bionx, MTS systems, USA). Details on the physicochemical analyses are reported in the supplementary methods.

### Critical-sized defects in sheep metatarsus

In this pilot study, three Ile de France sheep were deprived of food 24 hours before anesthesia. The technique used for creating a segmental defect in the metatarsus was according to a validated and previously described experimental model^48^. Briefly, the animals were anesthetized and treated with morphine hydrochloride (0.3 mg/kg, CDM Lavoisier, France) and oxytetracyline (20 mg/kg, Tenaline^®^ LA, Ceva, France) for analgesia and antibiotic prophylaxis. During the surgical procedure, general anesthesia was maintained by orotracheal intubation and inhalation of 3% isoflurane in oxygen. The heart and breathing rates were continuously monitored. After shaving and disinfecting the surgical site, a longitudinal incision was made to expose the medial surface of the left metatarsus. The customized, sterile surgical guide was positioned on the medial surface of the long bone and a surgical saw was inserted. A segmental critical-sized defect of 35 mm in length was created under constant saline irrigation to remove bone debris and prevent overheating. The defect was either left empty, treated with a customized 3D-printed scaffold alone or in combination with a vascular pedicle passing through. For the group that was only treated with the 3D-printed scaffold, the vascular plug was inserted into the main body of the scaffold and subsequently implanted. For the vascularized group, the pedicle was positioned in the center of the 3D scaffold and closed up with the vascular plug without performing vascular compression. The vascular pedicles in the non-vascularized group and in the empty defect retained in the natural anatomical position. Afterwards, the metatarsus was stabilized by using a seven-hole dynamic compression plate (LCP 3.5 L98, Depuy Synthes, Johnson & Johnson Medical, France) and four head-locking screws (diameter: 3.5 mm, length: 20 mm). The holes for the osteosynthesis screws were made by drilling them at appropriate positions with the surgical guide. To close the wound, subcutaneous tissues and skin were sutured in separate layers with resorbable sutures (Optime^®^ 4/0, Péters Medical, France). The limb was immobilized using a resin cast extending from nail to tibia that supported the weight with a splint for the entire period of the study. The sheep were allowed to bear weight immediately after the surgery. Post-operative pain was managed by administration of flunixine (1.5 mg/kg, Finadyne^®^, MSD Santé Animale, France). The animals were monitored by the clinical stuff of the facility for signs of infection during the entire period. Monthly CT angioscans checked for fractures and ‘vascualr patency’.

### Imaging and histological analyses

To monitor bone regeneration, CT angiscan were taken at day 0 (post-operative), 30, 60 and 90 using the same parameters as described for the pre-operative phase. After completing the CT scans at day 90, metatarsis were harvested and fixed in 4% formalin for 2 weeks at room temperature (Roti^®^-Histofix 4%, Carl Roth, France).

To obtain high-resolution images of the metatarsus at the endpoint of the study, explants were scanned using an *in vivo* cone-beam micro-computed tomography (microCT) scanner (Skyscan 1076, Bruker, Kontich, Belgium) during the fixation period. The X-ray tube was operated at 50 kV and 200 µA. Scans were recorded with 1° rotation step over 180° and exposure of 400 ms giving a resolution of 18 µm per pixel. The 3D reconstruction was performed using the accompanying software NRecon (Bruker, Kontich, Belgium). The percentage of bone volume + material volume over total volume (BV + MV/TV) was calculated in the selected region of interest corresponding to the diaphyseal defect and compared to the microCT of the 3D scaffold taken before implantation by the Skysan CT Analyzer (CTAn) software.

For histological examination, fixed samples were dehydrated in ascending grades of ethanol, immersed in xylol (Carl Roth) as intermedium and embedded in methyl methacrylate (Sigma Aldrich). Undecalcified thin ground sections were prepared in the longitudinal plane using the EXAKT cutting and grinding equipment (EXAKT Advanced Technologies, Norderstedt, Germany) according to the method established by Donath^49^. The sections were reduced to a thickness of 60 µm and stained with Levai-Laczko dye to differentiate between new bone, old bone and the 3D-printed scaffold^50^. For descriptive histological evaluation, the slides were scanned using an Olympus VS120 virtual slide microscope (Olympus, Tokyo, Japan) with a 20x objective.

### Statistical data analysis

Measurements of struts and pores sizes of the 3D scaffolds were performed using 20 readings on 3 different scaffolds. Mercury porosimetry, specific surface area and phase composition by XRD were determined on 3 samples. Compressive strength was measured on standard cylinders (n = 10/group). Data were expressed as average ± standard deviation. Since this is a pilot study with a limited number of animals, comparison between groups could not be performed. Data were graphed with GraphPad Prism software (GraphPad Software Inc., La Jolla, USA).

## Supplementary information

Supplementary materials.
