# Supplementary material for: Regeneration of segmental defects in metatarsus of sheep with vascularized and customized 3D-printed calcium phosphate scaffolds
Source: Sci Rep. 2020 Apr 27;10:7068. doi: 10.1038/s41598-020-63742-w (PMC7184564; doi:10.1038/s41598-020-63742-w)
Supplement: Supplementary file 1 — Supplementary materials. [file 41598_2020_63742_MOESM1_ESM.docx]

**Regeneration of segmental defects in metatarsus of sheep with vascularized and customized 3D-printed calcium phosphate scaffolds**

**Luciano Vidal, Carina Kampleitner, Stéphanie Krissian, Meadhbh Á Brennan, Oskar Hoffmann, Yago Raymond, Yassine Maazouz, Maria-Pau Ginebra, Philippe Rosset, Pierre Layrolle**

Online Data Supplement

Supplementary methods

Supplementary figures

Supplementary references

**Supplementary methods**

**Physicochemical analysis of the customized 3D calcium phosphate scaffolds**

The physicochemical properties of the 3D scaffolds were characterised by using different techniques. The dimensions of the structure, strand width and height, and pore size in the X/Y plane and in the Z direction were measured with an optical microscopy (Luxeo 4D Digital Stereozoom Microscope, Labomed Europe, The Netherlands) and an image analysis software (ImageJ)^1^. A total of 20 measurements on 3 different samples of each printing setup were carried out.

The microstructure of the scaffold was assessed by scanning electron microscopy (Phenom XL, Phenom-World B.V., ThermoFisher Scientific, The Netherlands). Therefore, the samples were coated with a thin carbon layer.

The phase composition of the scaffolds was assessed by X-ray diffraction (XRD, D8 Advance, Bruker, MA, U.S.A.) using a Cu Kα X-ray tube operated at 40 kV and 40 mA. Data were collected at a resolution of 0.02º/step over the 2θ range of 10º-80º with a counting time of 2 s/step. The resulting diffractogram was analysed with the reference intensity ratio method (EVA software, Bruker, U.S.A.) and the relative percentage of the crystalline structures of α-TCP (ICDD PDF 00-029-0359), CDHA (ICDD PDF 01-086-1201) and β-TCP (ICDD PDF 00-003-0681) were quantified.

The inter-strand porosity distribution (range from 0.006 to100 µm) was measured by mercury intrusion porosimetry (MIP) (AutoPore IV Micromeritics, USA). To obtain an analysis of the porosity percentage in the scaffold, this technique was combined with helium picnometry (AccuPyc 1330, Micromeritics, USA) and apparent density measurements, as described elsewhere^2^. Thus, quantifying the total porosity in the sample (P_TOT_) and assessing which of this percentage was attributable to the intra-strand porosity (<10 µm): nano-micro porosity (P_MICRO_); and to the inter-strand porosity (>10 µm): macro porosity (P_MACRO_).

The specific surface area (SSA) of the scaffold was measured with the Brunauer–Emmett–Teller (BET) method using a nitrogen adsorption surface area analyzer (ASAP 2020, Micromeritics, USA).

The mechanical properties of the scaffolds were analyzed using a universal testing machine (Bionx, MTS systems, USA). A total of 6 cylindrical samples of 20 mm height and 10 mm in diameter were used per condition. Samples were tested under a displacement rate control of 1 mm/min. The defined conditions were chosen following the ASTM C1424 – 99 standard.^3^ The load was applied in the Z direction, perpendicular to the printing plane. Prior to testing, the top layer of the scaffold was manually polished with P800 sandpaper to ensure the planarity and parallelism of the face with regards to the bottom face, and the samples were dried at 60 ºC overnight. The stress-strain curves obtained were analysed for the following parameters:

(1) The **ultimate compressive strength** (σ_UCS_) measured as the maximum compressive stress (MPa) that the sample withstand.

(2) The **Weibull modulus** (m), calculated by applying the Weibull statistical analysis to the ultimate compressive strength values (Eq. 1)

$$ln\left( ln\left( \frac{1}{P_{S}} \right) \right)= m\left( ln\left( \sigma_{UCS} \right)-ln\left( \sigma_{0} \right) \right)$$

Where P_s_ is the probability of survival and σ_0_ is a constant corresponding to the stress for which the 100% of the samples will not fail.

**Supplementary figures**


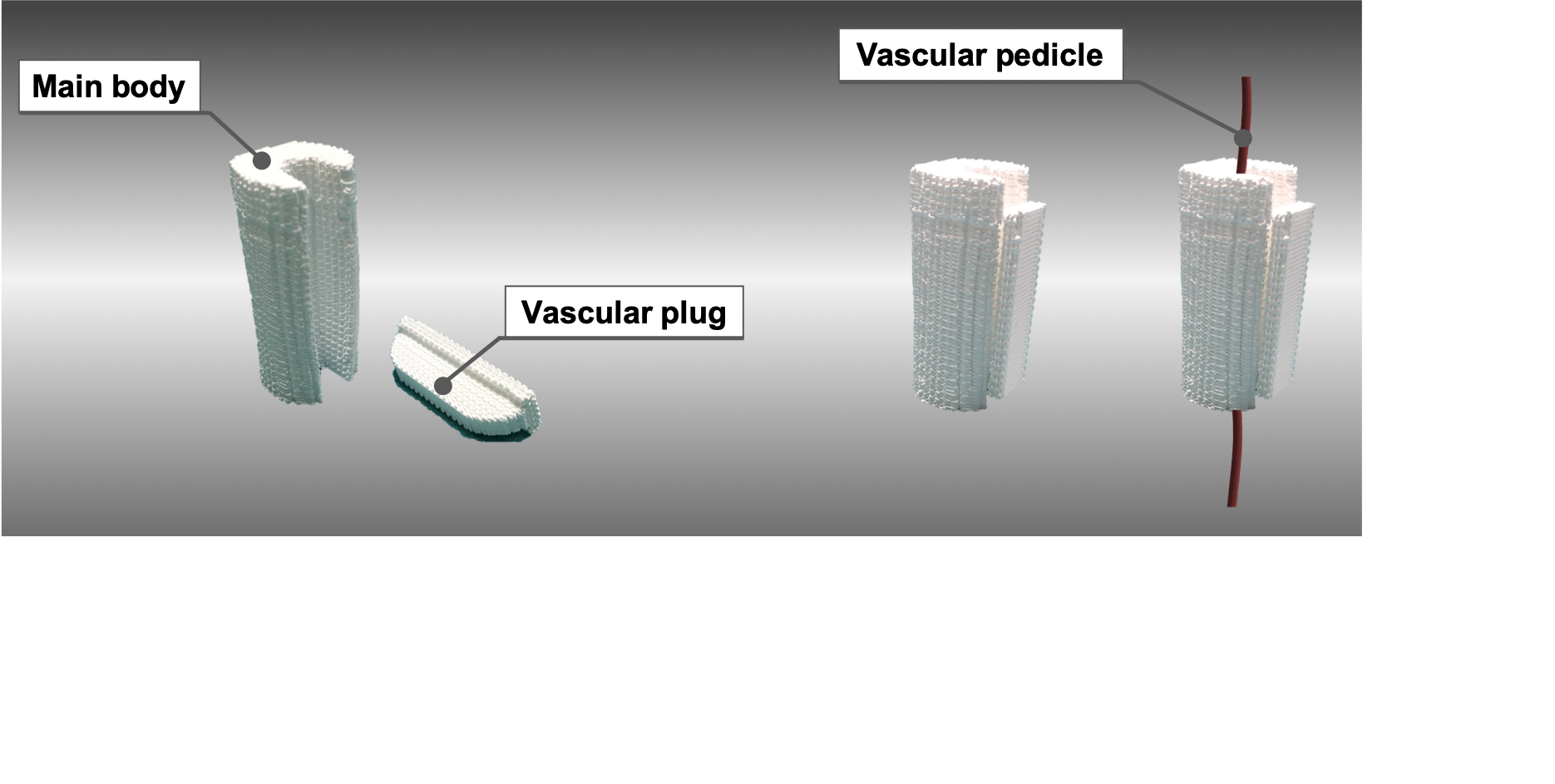


Supplementary Figure 1: **Schematic overview of the 3D-printed scaffold.** The 3D scaffold consisted of two parts: the main body with a central groove and the vascular plug. The diagram shows the two-part assembly including the axial vascular pedicle passing through.

*
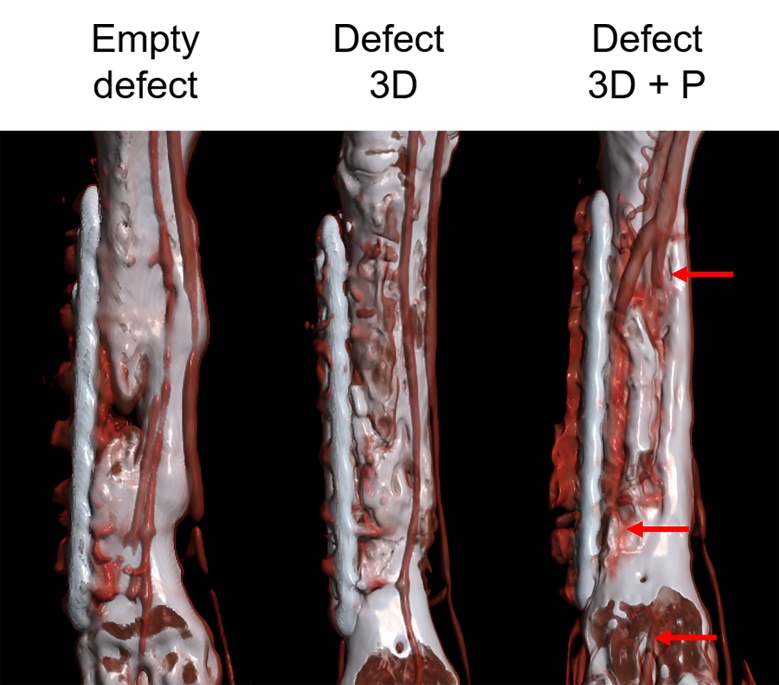
*

Supplementary Figure 2: **In vivo CT angioscans at D90 post surgery.** Treatment groups included the empty defect (control), defect filled with the 3D-printed scaffold (Defect 3D) and defect treated with the 3D-printed scaffold and the axial vascular pedicle (Defect 3D + P). Red arrows show the vascular patency of the pedicle.


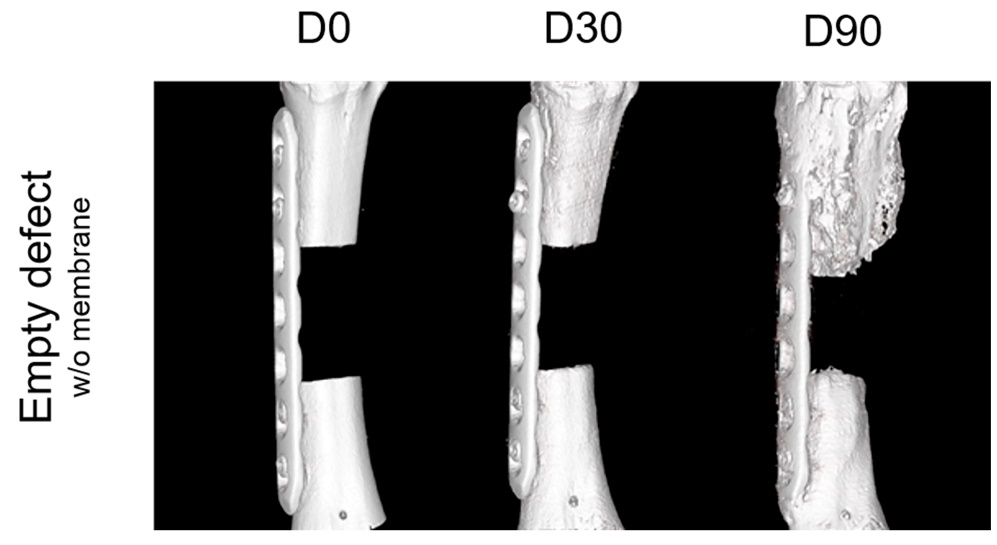


Supplementary Figure 3: **In vivo evaluation of bone healing in an empty defect after membrane resection.** A segmental mid-diaphyseal defect (length: 35 mm) was created in the metatarsus of sheep. To study the effect of the periosteum, the membrane was completely removed during the surgical procedure. CT scans are shown post-surgery (D0) and at 30 and 90 days of healing.

**Supplementary references**

1 Schindelin, J. *et al.* Fiji: an open-source platform for biological-image analysis. *Nat Methods* **9**, 676-682, doi:10.1038/nmeth.2019 (2012).

2 Pastorino, D., Canal, C. & Ginebra, M. P. Multiple characterization study on porosity and pore structure of calcium phosphate cements. *Acta Biomater* **28**, 205-214, doi:10.1016/j.actbio.2015.09.017 (2015).

3 ASTM C1424-99. Standard test method for monotonic compressive strength of advanced ceramics at ambient temperatures. (ASTM International, 1999).
